# The Record of an Active Year

**Published:** 1921-07-30

**Authors:** 


### The British Hospitals Association.

### THE RECORD OF AN ACTIVE YEAR.

The report of the Council of the British Hospitals Association, which was passed at the recent annual meeting at Manchester, deserves fuller notice than was possible in The Hospital when the report of the meeting appeared. In accordance with the revised rules, whereby the Council will eventually consist mainly of representatives of the Regional Committees, nine of these have already nominated members. The formation of these committees was the principal work of the year, and their value is apparent not merely in the provision of the considered opinion of the voluntary hospitals of the country, but also as the channel through which information received from Government Departments has been rapidly communicated. With these Departments the Council has been continually dealing.

The report specially refers to "the suggested amendment of the law (as it affects hospitals) in regard to the Workmen's Compensation Act, and to payments of income tax and legacy duty, especially in regard to relief from income tax on interest accrued due in respect of estates left by a testator, the winding-up of which, for various reasons, may take considerable time." The Council promises a further report on the important questions outstanding between the Ministry of Pensions and itself, and records that the terms obtained last year were the best which it was possible for the Ministry to pay. Negotiations are also proceeding between the Council and the Ministry of Labour, which, it will be remembered, held an inquiry on the Hours of Employment Bill in relation to nurses, porters, and other members of a hospital staff. In regard to the Unemployment Insurance Act, 1920, the report says that "the decision as to sisters is that unless it can be shown that the salary and emoluments of a sister bring her income up to £250 per annum, the sister is to be an insured person within the meaning of the Act." The Council is considering the advisability of an appeal, under Section 13 of the Act, to the High Court on the Minister's decision in regard to sisters and nurses.

To exempt hospitals from the regulations issued by the Home Office under the Dangerous Drugs Act, schemes were drafted for (1) hospitals with a pharmacist, (2) without, but with a medical officer, and (3) having neither, "each class of which may be exempted," subject to compliance with the necessary conditions in each case.

The report records that the issue of cheap tickets to patients going to and from convalescent homes is discontinued because the Minister of Transport (Command Paper No. 1148) has stated that since "the concession has only been continued in use during the war by two convalescent homes," local authorities and not the Ministry should bear the burden.

The Post Office was tackled over the new telephone rates, but the Council feel the force of the official argument that, since the principle is to charge each user with the cost of his service, preferential treatment to hospitals would entail a loss involving a surcharge on other users.

The Council reports also that the Library Fund attached to the original Association, which was raised by the late Sir Henry Burdett through the generosity of some of his friends, is to be transferred to the British Hospitals Association. The late Sir Henry Burdett and Mr. C. W. Thies were the trustees of this Fund, and the latter, as surviving trustee, is anxious to complete the matter. The funds, amounting roughly to £900 in cash and investments, together with the contents of the library, have been vested in three new trustees, and a library committee has been appointed. Its first members are the President (Sir A. Stanley), Mr. Wade-Deacon, Mr. W. H. Harper, Mr. Thomas Hayes, and Mr. Frank Hazell, who will hold office till the annual meeting in 1923. It will be remembered that the library has hitherto been lodged at 28 Southampton Street, Strand, where it remains at present.

